# Endogenous retroelement expression in modeled airway epithelial repair

**DOI:** 10.1016/j.micinf.2024.105465

**Published:** 2024-12-15

**Authors:** Stephanie Michael, Nicholas Liotta, Tongyi Fei, Matthew L. Bendall, Douglas F. Nixon, Nicholas Dopkins

**Affiliations:** Division of Infectious Diseases, Department of Medicine, Weill Cornell Medicine, New York, NY, USA

**Keywords:** Human endogenous retrovirus (HERV), Airway epithelium, Flagella, Endogenous retroelement (ERE), Long interspersed nuclear element type 1, (LINE1)

## Abstract

Cystic fibrosis (CF) is an autosomal recessive genetic disorder characterized by impairment of the CF transmembrane conductance regulator (CFTR) via gene mutation. CFTR is expressed at the cellular membrane of epithelial cells and functions as an anion pump which maintains water and salt ion homeostasis. In pulmonary airways of CF patients, pathogens such as *P. aeruginosa* and subsequent uncontrolled inflammation damage the human airway epithelial cells (HAECs) and can be life-threatening. We previously identified that inhibiting endogenous retroelement (ERE) reverse transcriptase can hamper the inflammatory response to bacterial flagella in THP-1 cells. Here, we investigate how ERE expression is sensitive to HAEC repair and toll-like receptor 5 (TLR5) activation, a primary mechanism by which inflammation impacts disease outcome. Our results demonstrate that several human endogenous retroviruses (HERVs) and long interspersed nuclear elements (LINEs) fluctuate throughout the various stages of repair and that TLR5 activation further influences ERE expression. By considering the impact of the most common CF mutation F508del/F508del on ERE expression in unwounded HAECs, we also found that two specific EREs, L1FLnI_2p23.1c and HERVH_10p12.33, were downregulated in CF-derived HAECs. Collectively, we show that ERE expression in HAECs is sensitive to certain modalities reflective of CF pathogenesis, and specific EREs may be indicative of CF disease state and pathogenesis.

## 1. Introduction

Cystic fibrosis (CF) is a recessive genetic disorder in which the CF transmembrane conductance regulator (CFTR) gene possesses a mutation that disrupts protein function [1,2]. The most common mutation, F508del/F508del, involves the loss of the amino acid phenylalanine at residue 508 [2]. Mucus accumulation, inadequate mucociliary clearance, and an acidic pH imbalance contribute to CF disease phenotypes of the lower pulmonary tract, however systemic pathologies are also observed in the gastrointestinal and endocrine systems [1]. A cocktail of elexacaftor-tezacaftor-ivacaftor (Trikafta) has been developed to improve upon existing pharmacotherapies [3,4] and may be approved for the ~90 % of CF patients in the United States that possess F508del/F508del mutations [5]. This development can provide relief to many patients, but the annual cost of Trikafta may not be accessible for all [6], and the sizeable portion of CF patients who do not possess F508del/F508del mutations will likely require alternative treatment strategies.

We aimed to better define the complex etiology of CF by investigating endogenous retroelement (ERE) expression in modeled human airway epithelial cell (HAEC) repair reflective of CF pathologies [7]. EREs comprise roughly ~41 % of the human genome [8,9] but their physiology has been understudied in comparison to canonical coding genes, and they have hence been overlooked in CF studies. EREs include human endogenous retroviruses (HERVs), long interspersed nuclear elements (LINEs), and short interspersed nuclear elements (SINEs), which constitute roughly ~8 %, ~13 %, and ~20 % of the human genome, respectively [8,9]. HERVs are the remnants of ancient retroviral infections which integrated into germ cells and have been passaged vertically for generations, however since their initial infection HERVs have accumulated multiple mutations and deletions which have rendered them replication incompetent and incapable of somatic reinfection [10]. Since their endogenization, these elements have contributed sequences of retroviral origin to the human genome with spatiotemporal activity that impacts human physiology [11]. LINEs are replication competent and autonomous retrotransposons that possess open reading frames for an endonuclease/reverse transcriptase protein and an RNA-binding protein [12]. SINEs are replication competent and nonautonomous retrotransposons that rely on the activity of LINE coding regions to retrotranspose. Collectively, EREs and other transposable elements (TEs) possess diverse roles in cellular biology [13] and immunity [14]. Emerging evidence finds EREs to be activated in response to pattern-associated molecular patterns (PAMPs) [15–19], encode immunostimulatory nucleic acid complexes [19–22], provide transcription factor binding sites that *cis*-regulate immune gene expression [23,24], regulate non-canonical immune gene variants through transposon exonization [25], and encode immunostimulatory antigens [26–28].

Previously, we observed that in response to bacterial flagella (FLA), the monocytic cell line THP-1 partially relies on the endogenous reverse transcription of EREs to acutely produce the proinflammatory cytokine tumor necrosis factor alpha (TNFα) [19]. *P. aeruginosa*, a life threatening infection to CF patients, possesses immunostimulatory FLA that exacerbates pathogenic immunity in CF patients through sustained activation of toll-like receptor 5 (TLR5) [29–31]. Viral infections which target airways also influence the activity of EREs in human epithelial cells [32–34], suggesting that their deregulated activity may be conserved during the stages of pathogenesis associated with immune activation. Collectively, ERE activity may be an understudied modulator of immunity and cellular processes during infections that compromise airway functionality of CF patients.

To calculate ERE expression at the RNA level in a model of CF, we analyzed transcriptomic data from a publicly available dataset with HAECs isolated from individuals with CF (F508del/F508del) and NCF controls (F508/F508) [7] using the bioinformatic pipeline Telescope [35]. Briefly, HAECs were cultured at the air-liquid interface and prepared from 7 individuals with CF and 6 NCF controls using the Muci-lAir™ platform. HAEC cultures were mechanically disrupted with an airbrush to replicate the effects of wounding. Following wounding, experimental groups were exposed to FLA to represent TLR5-dependent immune pathologies driven by *P. aeruginosa*. In analyzing this data, we quantify LINE and HERV RNAs in CF and NCF HAECs throughout the various stages of repair in conjunction with FLA. We identify two CF-specific EREs differentially expressed in normal HAECs, and we define numerous context-specific EREs differentially expressed in response to FLA exposure and throughout the various stages of HAEC repair. ERE transcription being influenced by CF status, particularly during delicate wounding states or vulnerable FLA exposure, warrants the exploration of specific ERE sequences with more precise and concerted analyses in CF.

## 2. Materials and methods

### 2.1. Locus-specific quantification of ERE expression with telescope

ERE quantification was performed with the bioinformatic pipeline Telescope as previously described [35,36]. Briefly, FASTQ files of bulk RNA sequencing performed on primary HAECs from a previous study [7] were accessed under the bioproject number “PRJNA525064” from the National Center for Biotechnology Information (NCBI) Sequence Read Archive (SRA). FASTQ files from PRJNA525064 were then aligned to the human genome build 38 (hg38) using STAR (v2.7.9.a) [37]. STAR alignment was performed using the custom parameters of “–outSAMstrandField intronMotif –outFilterMultimapNmax 200 –winAnchorMultimapNmax 200” to retain multimapping ERE reads. STAR aligned reads were further utilized to consider canonical gene expression patterns for 60,649 mRNAs. Following alignment to the hg38, we utilized the bioinformatic pipeline Telescope (v1.0.3) [35] to assign ERE reads with locus-specificity to a custom annotation of HERV and LINE elements available at https://github.com/mlbendall/telescope_annotation_db. The Telescope assign module was performed using the parameters “–theta_prior 200000 – max_iter 1000”.

### 2.2. Characterization of ERE expression profiles throughout HAEC repair stages

For the purpose of this study, only EREs possessing at least 2 reads within 10 % of the total samples were considered for downstream analysis. Of the 28,513 EREs described in the Telescope annotation, 2,649 passed this quality control threshold for consideration in downstream analyses. Differential expression analysis was performed between the ERE expression profiles of all HAEC repair conditions using DESEQ2 (v1.30.1) [38] with the parameters “parallel = T″ and “betaPrior = T”. Loci that possess a log2 fold change of >1 or < −1 and an adjusted p value (padj) of <0.05 were assumed to possess statistically significant differential expression between groups. Differentially expressed EREs were visualized with the R packages pheatmap (v1.0.12), ggVennDiagram (v1.2.2) [39] and EnhancedVolcano (v1.8.0) [40].

### 2.3. Statistics

All statistics were performed within DESEQ2 using a standard Wald’s Test with Benjamini-Hochberg correction of p values. Padj values were used for all comparisons. Padj values < 0.05 are referred to as significant.

## 3. Results

### 3.1. Differential expression of EREs throughout the stages of HAEC repair

**Fig. 1.**
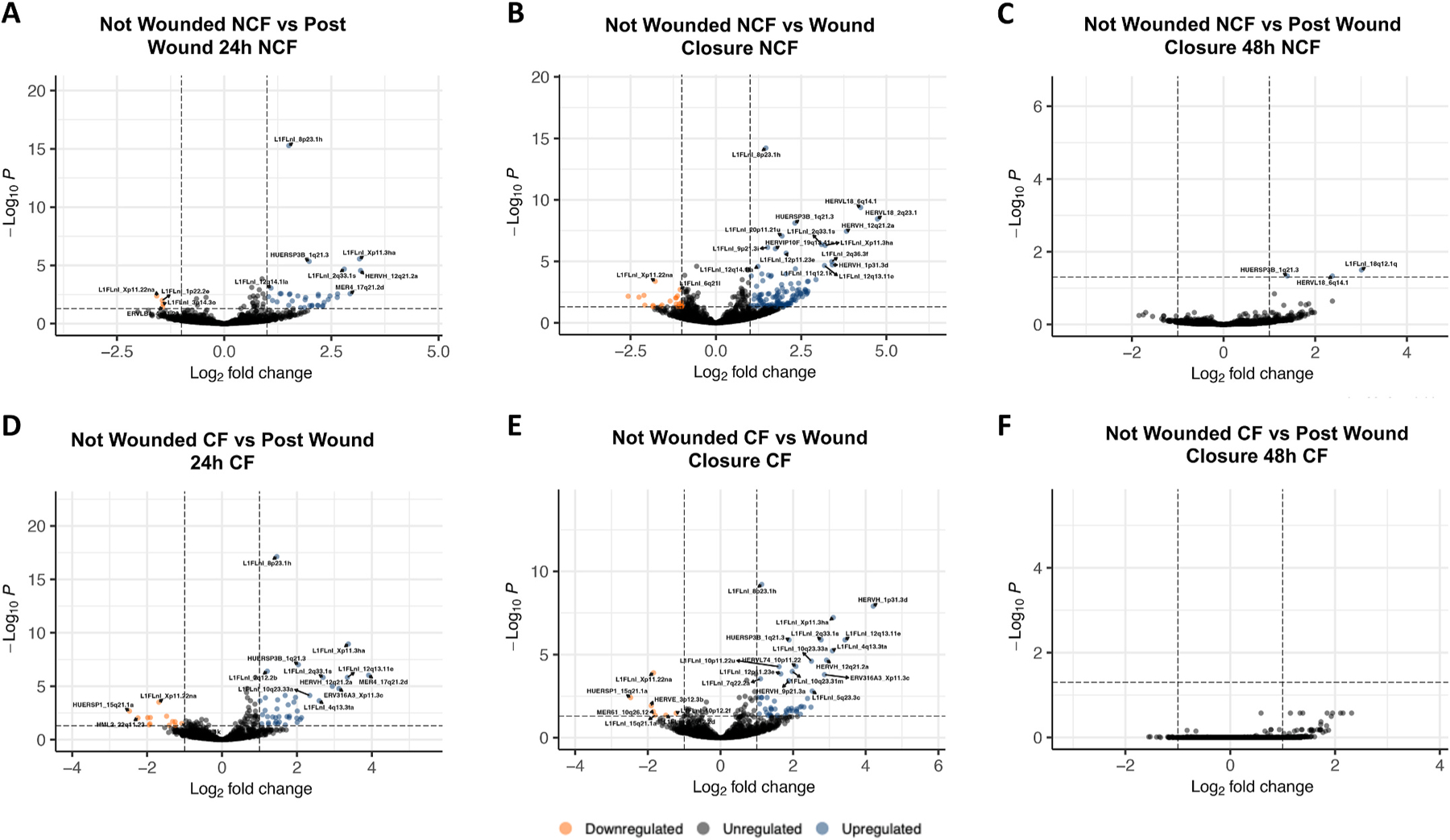
Differential expression of EREs throughout the stages of airway epithelial repair. Volcano plot demonstrates differential ERE expression between unwounded airway epithelial cells and airway epithelial cells 24 h after wounding from NCF-derived samples (**A**). Volcano plot demonstrates differential expression of EREs between unwounded airway epithelial cells and airway epithelial cells at wound closure from NCF-derived samples (**B**). Volcano plot demonstrates differential ERE expression between unwounded airway epithelial cells and airway epithelial cells 48 h after wound closure from NCF-derived samples (**C**). Volcano plot demonstrates differential ERE expression between unwounded airway epithelial cells and airway epithelial cells 24 h after wounding from CF-derived samples (**D**). Volcano plot demonstrates differential ERE expression between unwounded airway epithelial cells and airway epithelial cells at wound closure from CF-derived samples (**E**). Volcano plot demonstrates differential ERE expression between unwounded airway epithelial cells and airway epithelial cells 48 h after wound closure from CF-derived samples (**F**). All statistics were performed in DESEQ using the Wald’s Test. Adjusted p values were calculated using default parameters for a Benjamini-Hochberg correction. ERE reads were filtered to possess at least 2 reads in 10 % of the total samples for quality assurance. Differential expression was assumed if average fold change ≥1 or ≤ −1 and an adjusted p value of ≤0.05.

**Fig. 2.**
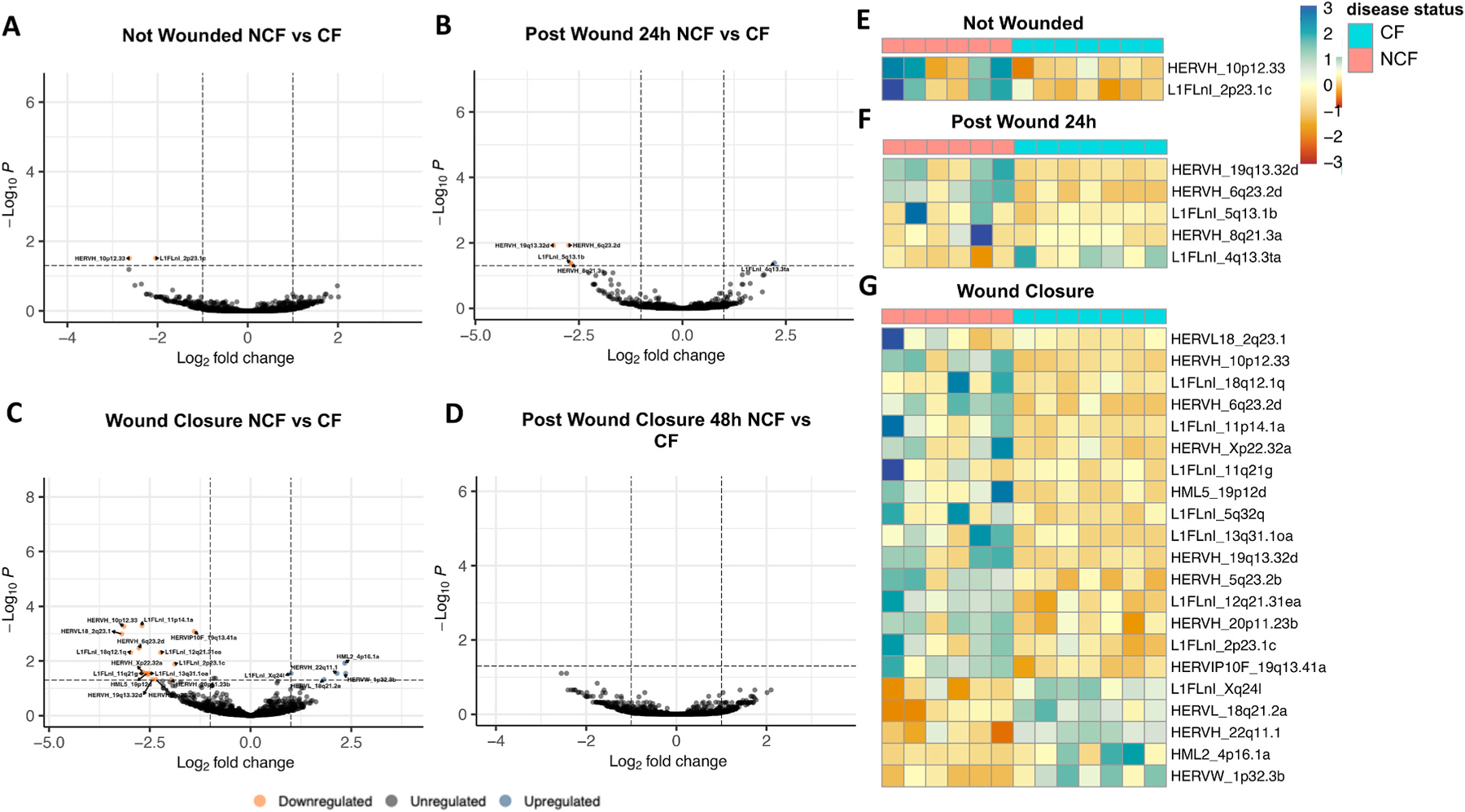
Cystic fibrosis status influences ERE expression during airway epithelial repair. Volcano plot demonstrates differential ERE expression between unwounded airway epithelial cells collected from NCF controls and patients with CF (**A**). Volcano plot demonstrates differential ERE expression between airway epithelial cells 24 h after wounding collected from NCF controls and patients with CF (**B**). Volcano plot demonstrates differential ERE expression between samples at wound closure of NCF and patients with CF (**C**). Volcano plot demonstrates differential ERE expression between samples 48 h after wound closure of NCF controls and patients with CF (**D**). Heatmap demonstrates the normalized abundance of differentially expressed ERE transcripts per sample of unwounded airway epithelium across NCF controls (red) and patients with CF (blue) (**E**). Heatmap demonstrates the normalized abundance of differentially expressed ERE transcripts per sample of airway epithelium 24 h after wounding across NCF controls (red) and patients with CF (blue) (**F**). Heatmap demonstrates the normalized abundance of differentially expressed EREs per sample of airway epithelium at wound closure across NCF controls (red) and patients with CF (blue) (**G**). All statistics were performed in DESEQ using the Wald’s Test. Adjusted p values were calculated using default parameters for a Benjamini-Hochberg correction. ERE reads were filtered to possess at least 2 reads in 10 % of the total samples for quality assurance. Differential expression was assumed if average fold change ≥1 or ≤ −1 and an adjusted p value of ≤0.05. (For interpretation of the references to colour in this figure legend, the reader is referred to the Web version of this article.)

We sought to identify signatures of ERE activity that define the discrete stages of HAEC repair. Applying the same preprocessing filtering criterion mentioned above, we observed an abundantly and significantly different profile of EREs active during the stages of repair. Compared to unwounded HAECs of NCF controls, HAECs 24 h after wounding displayed 4 significantly downregulated and 35 significantly upregulated EREs (Fig. 1a), while HAECs at wound closure displayed 22 significantly downregulated and 126 significantly upregulated EREs (Fig. 1b). Compared to unwounded HAECs from patients with CF, HAECs 24 h after wounding displayed 13 significantly downregulated and 42 significantly upregulated EREs (Fig. 1d), while HAECs at wound closure displayed 7 significantly downregulated and 25 significantly upregulated EREs (Fig. 2e). In HAECs collected from NCF controls and patients with CF, HAECs 48 h after wound closure were most similar to their unwounded counterparts, with 3 EREs significantly upregulated between NCF controls (Fig. 1c) and no significant differential expression of EREs detected between cells from individuals with CF (Fig. 1f). Briefly, these results indicate significant differential expression patterns of EREs during wounding repair stages, which correct upon completion of wound healing.

### 3.2. Cystic fibrosis status influences ERE expression during HAEC repair

To accurately capture the expression profiles of HAEC samples from NCF controls and patients with CF during different stages of repair, we filtered all transcript reads to remove EREs with fewer than 2 reads in at least 10 % of samples. Across all samples, 2649 EREs met this preprocessing criterion. Of these, 2 EREs from unwounded HAECs (Fig. 2a) and 4 EREs from HAECs 24 h after wounding were significantly downregulated in CF-derived samples (Fig. 2b), and 1 ERE from HAECs 24 h after wounding was significantly upregulated in CF-derived samples (Fig. 2b). In HAECs at wound closure, 15 EREs were found to be significantly downregulated and 5 EREs significantly upregulated in CF-derived samples (Fig. 2c). No significant differences in ERE expression were identified between HAECs isolated from NCF- and CF-derived samples 48 h after wound closure (Fig. 2d), indicating a potential acute activation of ERE expression that subsides over time. Significant differences identified between NCF- and CF-derived samples during the wounding conditions are conveyed with per sample abundances (Fig. 2e–g). Overall, the profile of ERE transcripts in HAECs appears consistently differentiated across the processes of repair between NCF- and CF-derived samples.

### 3.3. Flagella impacts ERE expression in primary HAECs

**Fig. 3.**
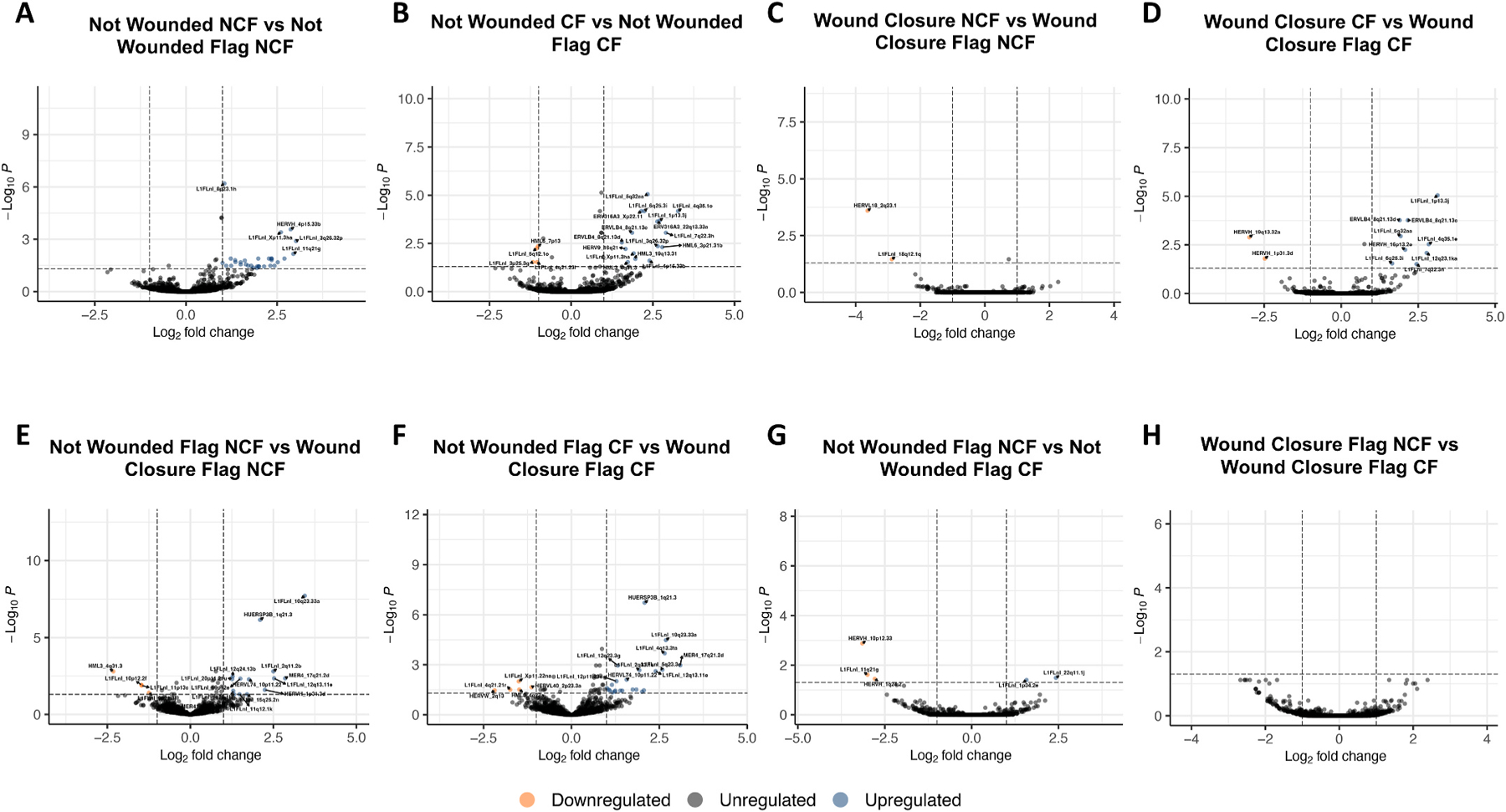
Flagella impacts ERE expression in primary airway epithelial cells. Volcano plot demonstrates differential expression of EREs between unwounded airway epithelial cells and unwounded airway epithelial cells stimulated with FLA from NCF-derived samples (**A**). Volcano plot demonstrates differential expression of EREs between unwounded airway epithelial cells and unwounded airway epithelial cells stimulated with FLA from CF-derived samples (**B**). Volcano plot demonstrates differential expression of EREs between airway epithelial cells at wound closure and airway epithelial cells at wound closure stimulated with FLA from NCF-derived samples (**C**). Volcano plot demonstrates differential expression of EREs between airway epithelial cells at wound closure and airway epithelial cells at wound closure stimulated with FLA from CF-derived samples (**D**). Volcano plot demonstrates differential expression of EREs between unwounded airway epithelial cells stimulated with FLA and airway epithelial cells at wound closure stimulated with FLA from NCF-derived samples (**E**). Volcano plot demonstrates differential expression of EREs between unwounded airway epithelial cells stimulated with FLA and airway epithelial cells at wound closure stimulated with FLA from CF-derived samples (**F**). Volcano plot demonstrates differential expression of EREs between unwounded airway epithelial cells stimulated with FLA from NCF-derived samples and unwounded airway epithelial cells stimulated with FLA from CF-derived samples (**G**). Volcano plot demonstrates differential expression of EREs between airway epithelial cells at wound closure stimulated with FLA from NCF-derived samples and unwounded airway epithelial cells at wound closure stimulated with FLA from CF-derived samples (**H**). All statistics were performed in DESEQ using the Wald’s Test. Adjusted p values were calculated using default parameters for a Benjamini-Hochberg correction. ERE reads were filtered to possess at least 2 reads in 10 % of the total samples for quality assurance. Differential expression was assumed if average fold change ≥1 or ≤ −1 and an adjusted p value of ≤0.05.

Next, we explored ERE expression signatures potentially specific to FLA exposure in unwounded and post-wound conditions. We generally observed a consistent upregulation of EREs with exposure to FLA. FLA stimulation among unwounded HAECs induced a significant upregulation of 32 EREs in NCF-derived samples (Fig. 3a) and a significant downregulation of 4 EREs and a significant upregulation of 16 EREs in CF-derived samples (Fig. 3b). FLA stimulation among HAECs at wound closure induced a significant downregulation of 2 EREs in NCF-derived samples (Fig. 3c) and a significant downregulation of 2 EREs and a significant upregulation of 9 EREs in CF-derived samples (Fig. 3d). Within NCF-derived samples, HAECs at wound closure stimulated with FLA displayed 4 significantly downregulated and 14 significantly upregulated EREs compared to unwounded HAECs stimulated with FLA (Fig. 3e). CF-derived samples, on the other hand, yielded 5 significantly downregulated and 23 significantly upregulated EREs in HAECs at wound closure stimulated with FLA compared to unwounded HAECs stimulated with FLA (Fig. 3f). Unwounded FLA-stimulated HAECs from CF-derived samples displayed 3 significantly downregulated and 2 significantly upregulated EREs compared to NCF-derived samples (Fig. 3g). FLA-stimulated HAECs at wound closure showed no significant difference in ERE expression between NCF- and CF-derived samples (Fig. 3h).

### 3.4. Sequential ERE expression changes throughout the stages of airway epithelium repair

**Fig. 4.**
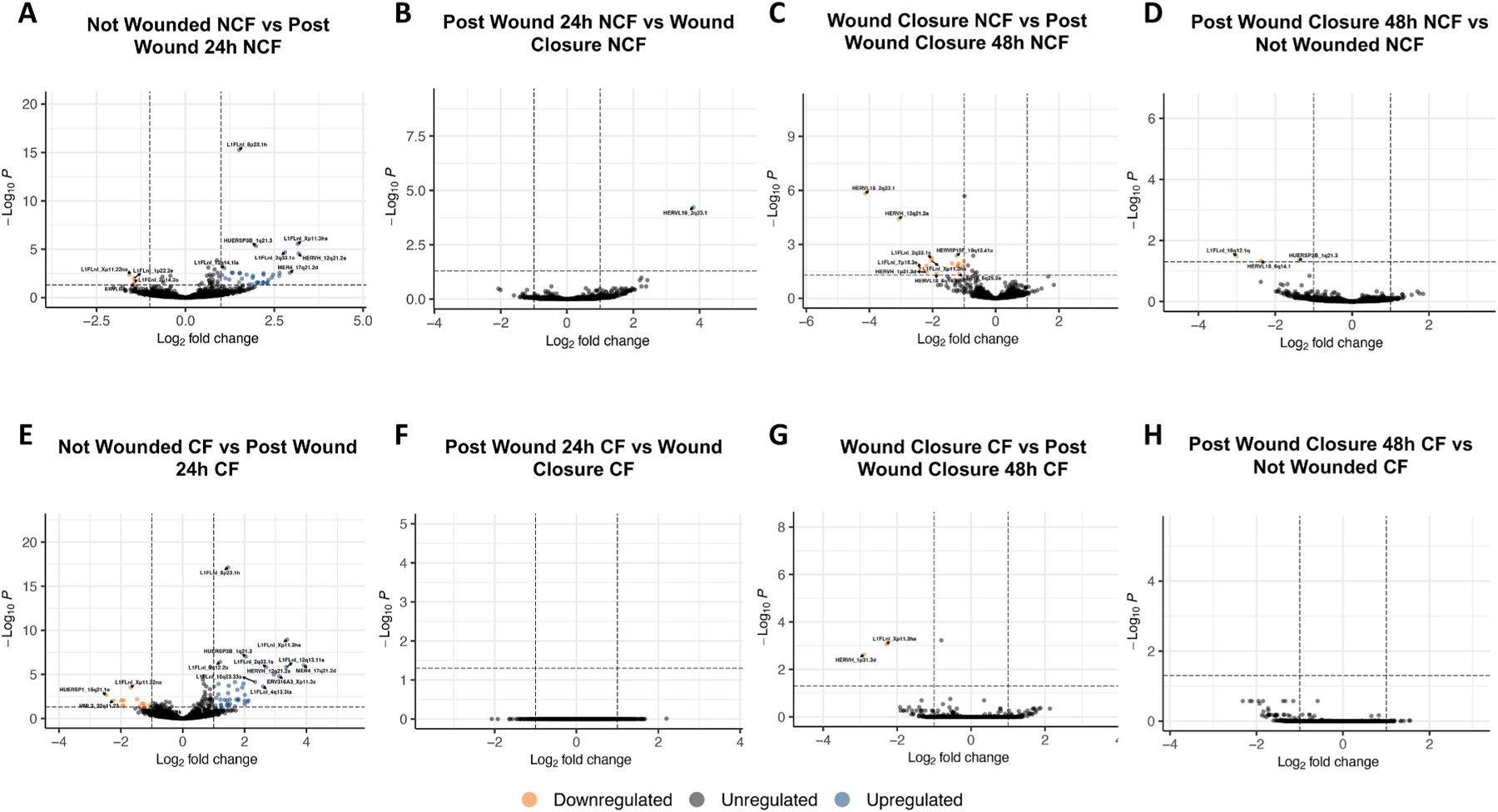
Sequential ERE expression changes throughout the stages of airway epithelium repair. Volcano plot demonstrates differential expression of EREs between unwounded airway epithelial cells and airway epithelial cells 24 h after wounding within NCF-derived control samples (**A**). Volcano plot demonstrates differential expression of EREs between airway epithelial cells 24 h after wounding and at wound closure within NCF-derived control samples (**B**). Volcano plot demonstrates differential expression of EREs between airway epithelial cells at wound closure and 48 h post wound closure within NCF-derived control samples (**C**). Volcano plot demonstrates differential expression of EREs between airway epithelial cells 48 h post wound closure and unwounded within NCF-derived control samples (**D**).Volcano plot demonstrates differential expression of EREs between unwounded airway epithelial cells and airway epithelial cells 24 h after wounding within CF-derived samples (**E**). Volcano plot demonstrates differential expression of EREs between airway epithelial cells 24 h after wounding and at wound closure within CF-derived samples (**F**). Volcano plot demonstrates differential expression of EREs between airway epithelial cells at wound closure and 48 h post wound closure within CF-derived samples (**G**). Volcano plot demonstrates differential expression of EREs between airway epithelial cells 48 h post wound closure and unwounded within CF-derived samples (**H**). All statistics were performed in DESEQ using the Wald’s Test. Adjusted p values were calculated using default parameters for a Benjamini-Hochberg correction. ERE reads were filtered to possess at least 2 reads in 10 % of the total samples for quality assurance. Differential expression was assumed if average fold change ≥1 or ≤ −1 and an adjusted p value of ≤0.05.

Finally, we investigated ERE expression signatures occurring sequentially throughout the stages of HAEC wounding and repair to determine temporal reflections in retroelement expression. Compared to not wounded samples, wounding of HAECs induced significant differential expression of numerous EREs in NCF-derived samples (Fig. 4a). By comparing the expression of samples at wound closure to those post wounding in NCF-derived samples, we then find 1 ERE differentially expressed (Fig. 4b). 48 h after wound closure however, numerous EREs then are downregulated in comparison to the initial stages of wound closure in NCF-derived samples, including the ERE initially upregulated by the previous stage of repair (Fig. 4c). Comparisons between post wounding and not wounded samples derived from NCF controls then demonstrate the differential activity of 3 EREs (Fig. 4d). Compared to not wounded samples, wounding of HAECs induced significant differential expression of numerous EREs in CF-derived samples (Fig. 4e). By comparing the expression of samples at wound closure to those post wounding in CF-derived samples, we then see not differential activity in EREs (Fig. 4f). At 48 h post wound closure however, only 2 EREs are then are downregulated in comparison to the initial stages of wound closure in CF-derived samples (Fig. 4g). Comparisons between post wounding and not wounded samples derived from CF show no differential ERE activity (Fig. 4d).

## 4. Discussion

In an effort to better characterize genetic sequences implicated in CF pathology and their functions, we explored how ERE expression may participate in HAEC repair in samples collected from individuals with CF. ERE expression was quantified via sequenced RNA from HAECs of NCF- and CF-derived samples during four stages of healing. Across two of the wounding conditions, 24 h post wound and wound closure, we observed a general pattern of ERE downregulation in CF-derived samples compared to NCF (Fig. 2). This distinction persists between unwounded samples, with 2 downregulated ERE loci that are intergenic, HERVH_10p12.33 and L1FLnI_2p23.1c (Fig. 2a and e), suggesting that CF mutational status alone may downregulate their expression. Canonical gene analysis revealed similar patterns of predominant and widespread downregulation in CF-derived HAECs unwounded, 24 h post wound, and at wound closure when compared to NCF-derived HAECs (Supplemental Fig. 1). It is unclear whether EREs are also downregulated by an identical or merely convergent process, and their potential biological implications in CF-derived samples warrant further investigation.

Interestingly, HERVH_10p12.33 and L1FLnI_2p23.1c in CF-derived samples are expressed at similar levels to their NCF counterparts 24 h after wounding (Fig. 2f), but they both appear significantly downregulated at wound closure (Fig. 2g). Although no ERE loci were found to be significantly differentially expressed between NCF- and CF-derived samples 48 h after wound closure, downregulation of HERVH_10p12.33 is near significant at this stage (p = 0.079) (Fig. 2d). These results reflect the observed pattern of a transcriptomic ERE profile that is most different between NCF- and CF-derived samples at wound closure, with 15 EREs significantly downregulated and 5 significantly upregulated in CF-derived samples (Fig. 2c).

We observed a difference in the expression patterns between unwounded samples and samples 48 h after wound closure, when healing is expected to be mostly complete. Unlike CF-derived HAECs which were never wounded, CF-derived HAECs which had undergone both wounding and wound closure demonstrated no significant difference in ERE expression against their NCF counterparts (Fig. 2d). A delayed return to normalcy for EREs is mirrored by canonical gene analysis, with post-wound-closure expression levels for certain genes to still be in the process of rising to their respective unwounded levels (Supplementary Fig. 1). Altogether, our observations indicate a pattern of ERE expression dependent on CF status at the RNA level and have identified 2 EREs downregulated in CF-derived samples with expression patterns potentially attributed to the mutational status of the CFTR gene.

To understand how wounding-specific transcriptomes may be subject to change by CF status during the 90-h course of HAEC repair, we evaluated RNA read counts from the 3 wounding conditions against read counts from unwounded HAECs. In both NCF- and CF-derived samples, we observed a broad and significant upregulation of ERE loci during the stages of repair. At wound closure, the expression of some elements appears dampened by CF status, with transcript counts several times lower in CF-derived HAEC at wound closure than NCF-derived HAEC at wound closure compared to their unwounded counterparts (Fig. 1b and e). This is confirmed by our earlier comparison of ERE expression during wounding conditions of NCF- and CF-derived HAECs, which found a comparatively lower transcription of ERE loci in CF-derived HAECs, particularly at wound closure (Fig. 2c). Finally, 3 ERE loci (HUERSP3B_1q21.3, HERVL18_6q14.1, and L1FLnl_18q12.1q) appear significantly upregulated in NCF-derived HAECs 48 h after wound closure compared to unwounded HAECs, but the expression of these loci 48 h after wound closure appears indistinguishable from unwounded HAECs in CF-derived HAECs (Fig. 1c and f). None of the three loci are differentially expressed in unwounded CF-derived HAECs compared to unwounded NCF-derived HAEC (Fig. 2a), which signifies a differential expression of EREs during the final stages of HAEC repair. Collectively, the trend of differentially expressed ERE RNAs primarily being upregulated during the various stages of wound healing is reflected in the canonical coding gene profiles of the same samples (Supplementary Fig. 2). In the context of wounding, ERE expression may be important in the innate immune processes that coordinate immune cell chemotaxis, which then facilitates reconstructive inflammation as demonstrated by microbe-induced ERE activity in murine keratinocytes [15]. Taken together, a predominant upregulation of ERE and canonical gene loci across the three wounded HAEC conditions indicates a substantial mobilization of differential transcriptomic profiles to facilitate the epithelial repair process, most notably at a level slightly dampened by CF status.

We have previously observed that in response to bacterial FLA, the monocytic cell line THP-1 partially relies on the endogenous reverse transcription of EREs to acutely produce the proinflammatory cytokine tumor necrosis factor alpha [19]. For this purpose, we investigated whether the activation of primary HAECs with FLA impacts ERE activity at the RNA level, potentially contributing to the inflammatory etiology of CF [29–31]. In investigating whether HAEC ERE expression is sensitive to the stages of wound repair, we found that FLA exposure influences the differential expression of multiple EREs, primarily upregulating expression, in unwounded HAECs collected from CF and NCF donors (Fig, 3a-b). At wound closure, FLA did not demonstrate a notable effect on NCF-derived HAECs, only downregulating 2 EREs, but induced the significant upregulation of 9 EREs in CF-derived HAECs (Fig, 3c-d). Although EREs responsive to FLA in these models are few, their observed differences could indicate a modulation of ERE activity by mutational CFTR status in HAECs stimulated with FLA. In accordance with previous comparisons, the patterns of differential expression for ERE RNAs stimulated with FLA is reflected in the canonical coding gene profiles of the same samples (Supplementary Fig. 3). This model suggests that CF samples primarily at the wound closure stage transcriptionally differ from NCF samples for both ERE and canonical gene RNAs. Collectively, the expression of EREs at mucosal barriers is likely sensitive to microbial byproducts as previously shown [15,16], and further research on the physiological implications of this activity is required.

Some limitations pertain to quantification of EREs performed in this study. Briefly, ERE RNAs are proportionally less abundant than canonical coding gene mRNAs [41,42], complicating their detection and quantification from RNA sequencing approaches [43]. To compensate for accurately quantifying their expression, we stringently filtered against lowly abundant transcripts, resulting in 2649 being considered for comparisons. Compared to the 60,649 from STAR outputs, the number of differentially expressed EREs are fewer. Further studies utilizing sequencing technologies that improve upon ERE recovery and deconvolution, such as long read sequencing [44], can improve upon the characterization of lowly abundant sequences that may possess physiological roles in CF and other conditions. Additionally, this retrospective analysis of existing sequencing datasets was performed on *ex vivo* samples and lacks biological validation. Therefore, the extent by which these changes in ERE expression profiles translate to *in vivo* HAEC expression remain undescribed. Furthermore, this data was compiled using data provided from a single study in which the demographic metadata pertaining to HAEC donors was not retrievable for consideration in this analysis. Due to which, the impact of confounding factors (e.g., diet and smoking status) on ERE expression in the context of HAEC repair could not be ascertained and requires further research.

In conclusion, we identify 2 intergenic EREs, HERVH_10p12.33 and L1FLnI_2p23.1c, whose expression in HAECs is inversely correlated with the most common mutation of the CTFR gene (Supplementary Fig. 4). Whether they possess any role in mucosal homeostasis or whether their expression impacts organismal physiology requires further study. We also describe that certain EREs are sensitive to the stages of airway epithelium repair modeled in primary HAECs from CF and NCF donors. As microbial assimilation, host immunity, and cell growth are all integral to mucosal homeostasis, understudied etiological components such as EREs pose a significant, untapped resource to potentially uncover the underlying physiology of host-microbe interactions.

## Supplementary Material

1
