# Supplementary material for: Endogenous retroelement expression in modeled airway epithelial repair
Source: Microbes Infect. Author manuscript; Available in PMC 2025 Aug 10. (PMC12167390; doi:10.1016/j.micinf.2024.105465)
Supplement: 1 [file NIHMS2043793-supplement-1.pdf]

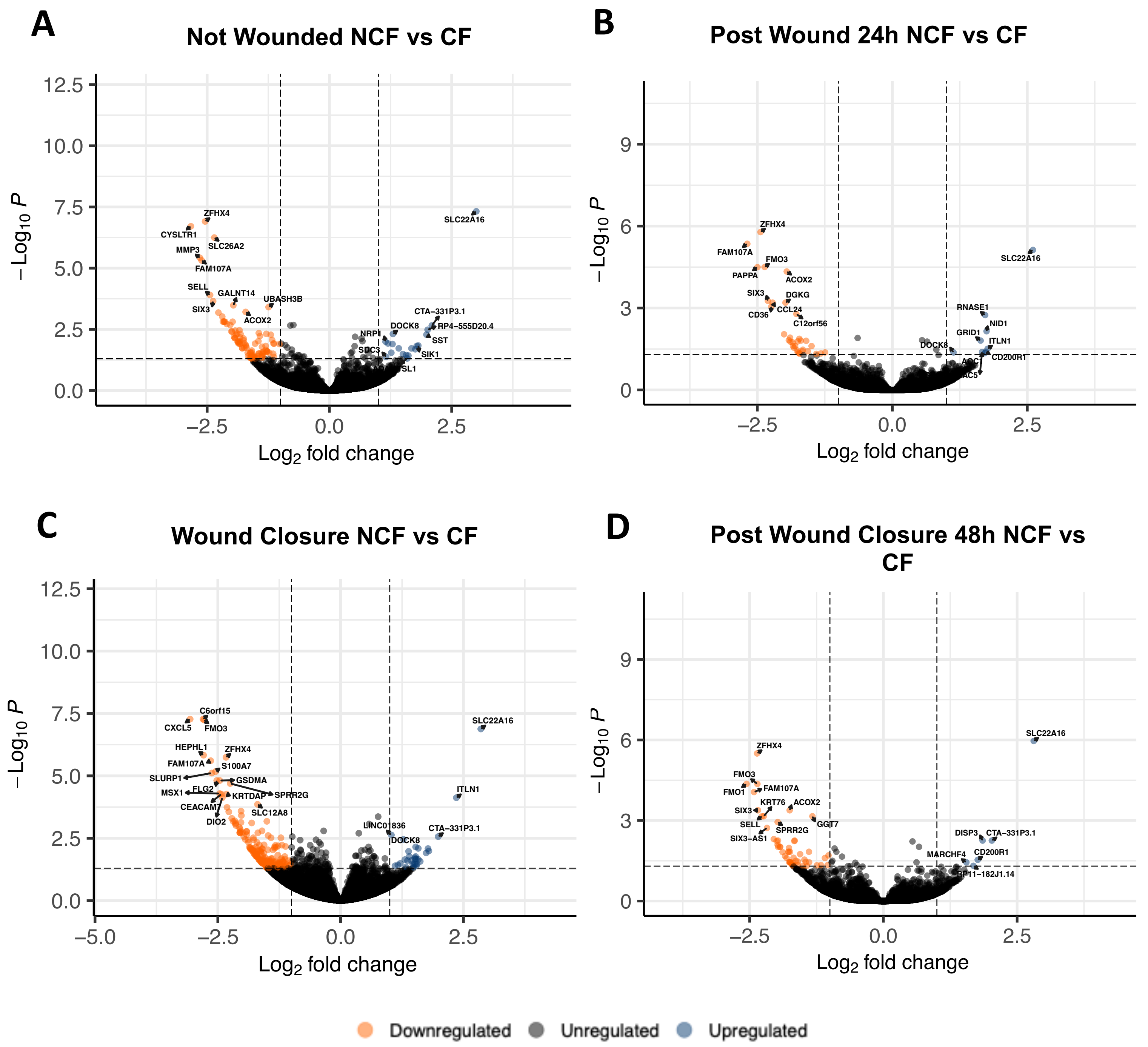
A
Not Wounded NCF vs CF
B
Post Wound 24h NCF vs CF
C
Wound Closure NCF vs CF
D
Post Wound Closure 48h NCF vs CF
−Log10 P
Log2 fold change
SLC22A16
ZFHX4
FAM107A
Downregulated
Unregulated
Upregulated

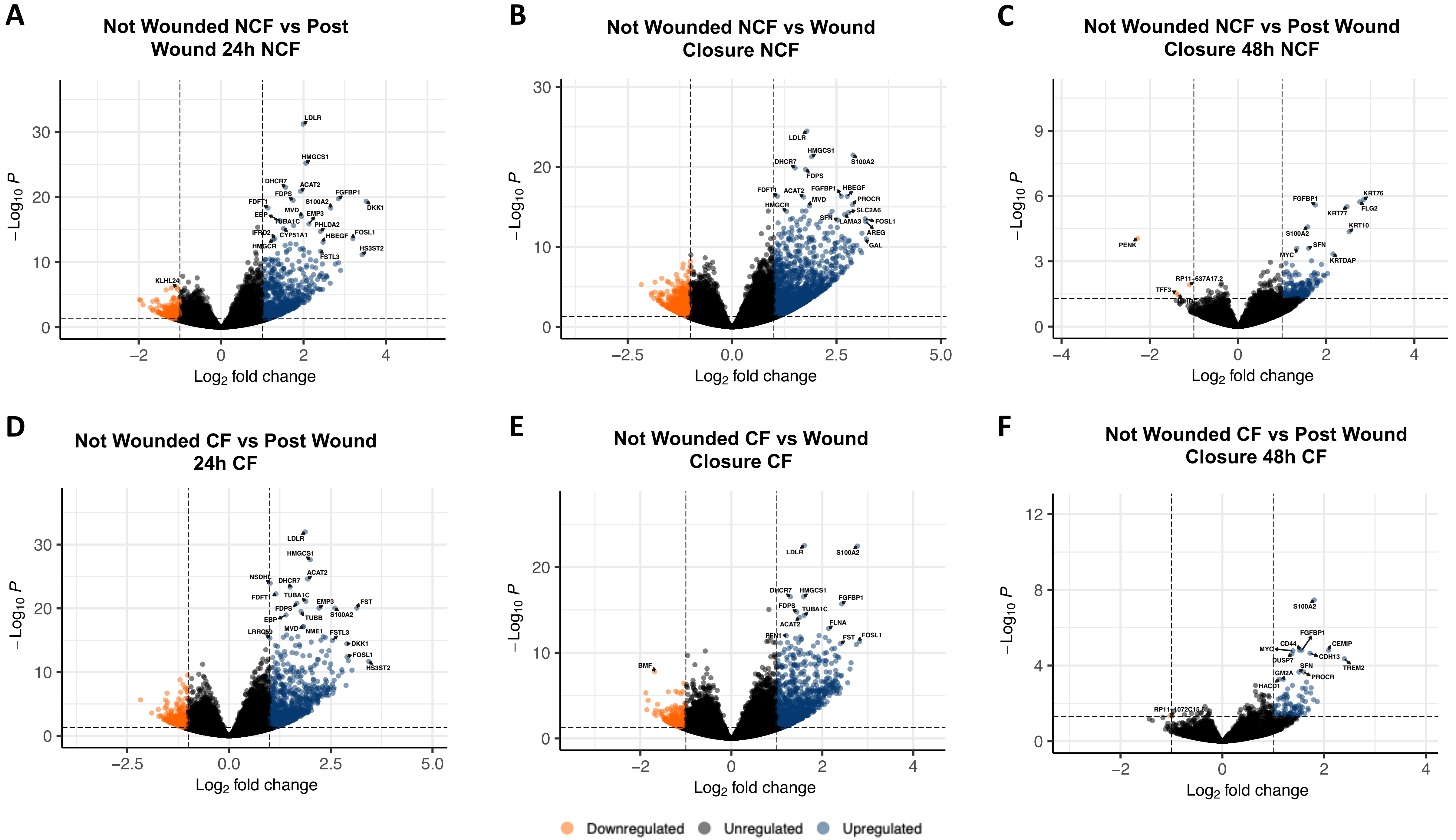
A
Not Wounded NCF vs Post Wound 24h NCF
LDLR
HMGCS1
DHCR7
ACAT2
FDPS
FGFBP1
FDFT1
S100A2
DKK1
MVD
EBP
EMP3
TUBA1C
PHLDA2
IFRD2
CYP51A1
HBEGF
FOSL1
HMGCR
HS3ST2
FSTL3
KLHL24
−Log10 P
Log2 fold change
B
Not Wounded NCF vs Wound Closure NCF
LDLR
HMGCS1
S100A2
DHCR7
FDPS
FDFT1
ACAT2
FGFBP1
HBEGF
MVD
PROCR
HMGCR
SLC2A6
SFN
LAMA3
FOSL1
AREG
GAL
−Log10 P
Log2 fold change
C
Not Wounded NCF vs Post Wound Closure 48h NCF
KRT76
FGFBP1
KRT77
FLG2
KRT10
S100A2
PENK
SFN
MYC
KRTDAP
RP11-637A17.2
TFF3
−Log10 P
Log2 fold change
D
Not Wounded CF vs Post Wound 24h CF
LDLR
HMGCS1
ACAT2
NSDHL
DHCR7
FDFT1
TUBA1C
EMP3
FST
FDPS
S100A2
EBP
TUBB
MVD
NME1
FSTL3
LRRC59
DKK1
FOSL1
HS3ST2
−Log10 P
Log2 fold change
E
Not Wounded CF vs Wound Closure CF
LDLR
S100A2
DHCR7
HMGCS1
FGFBP1
FDPS
TUBA1C
ACAT2
FLNA
PFN1
FST
FOSL1
BMF
−Log10 P
Log2 fold change
F
Not Wounded CF vs Post Wound Closure 48h CF
S100A2
FGFBP1
CEMIP
CD44
MYC
CDH13
DUSP7
TREM2
SFN
GM2A
PROCR
HACD1
RP11-1072C15.6
−Log10 P
Log2 fold change
Downregulated
Unregulated
Upregulated

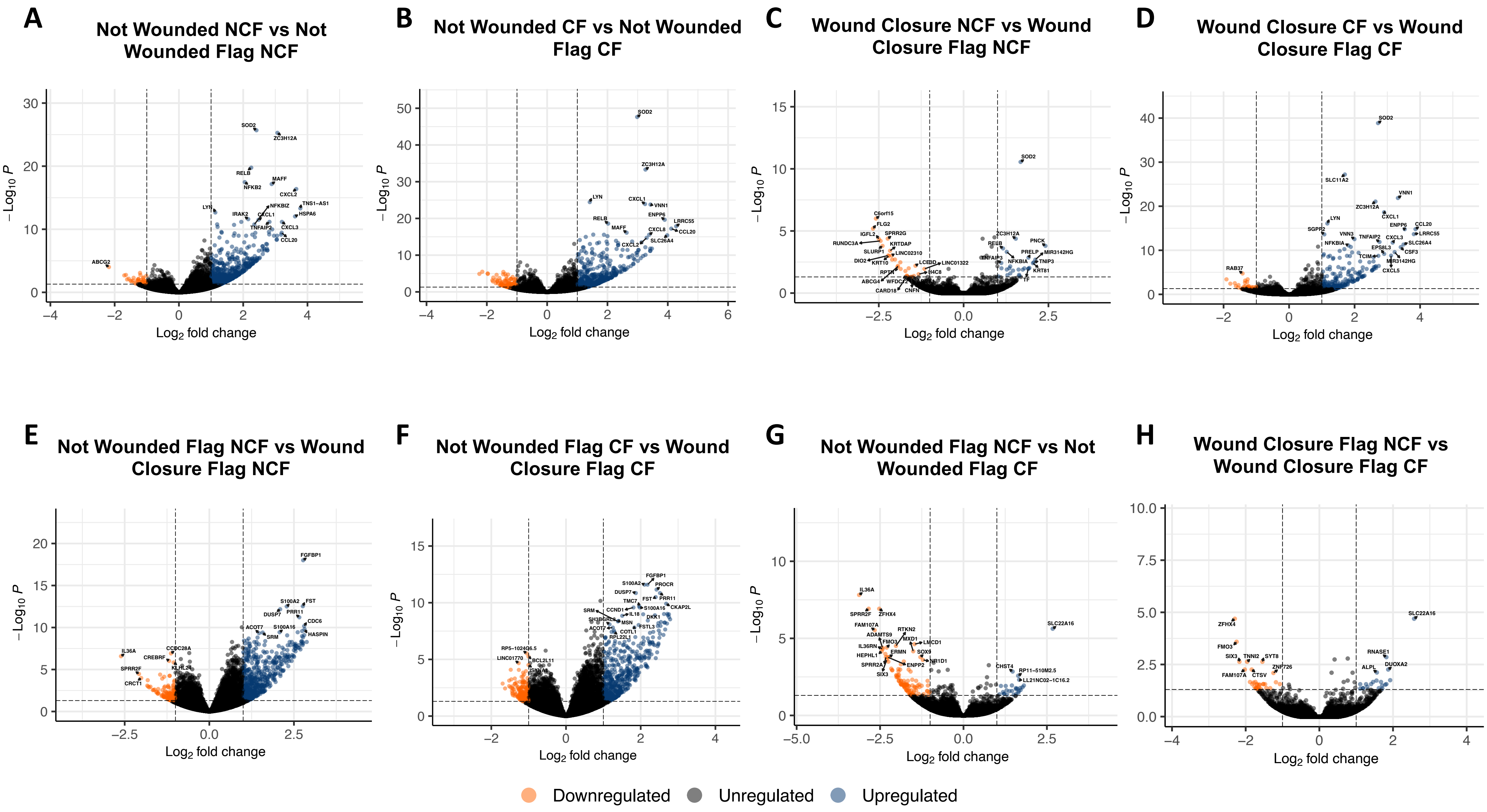
A
Not Wounded NCF vs Not Wounded Flag NCF
SOD2
ZC3H12A
RELB
NFKB2
MAFF
CXCL2
NFKBIZ
TNS1−AS1
LYN
IRAK2
CXCL1
HSPA6
TNFAIP2
CXCL3
CCL20
ABCG2
B
Not Wounded CF vs Not Wounded Flag CF
SOD2
ZC3H12A
LYN
CXCL1
VNN1
RELB
ENPP6
LRRC55
MAFF
CXCL8
CCL20
CXCL2
SLC26A4
C
Wound Closure NCF vs Wound Closure Flag NCF
SOD2
C6orf15
FLG2
IGFL2
SPRR2G
RUNDC3A
KRTDAP
SLURP1
LINC02310
DIO2
KRT10
LINC01322
RPTN
ABCG4
WFDC12
CARD18
CNFN
ZC3H12A
RELB
PNCK
PRELP
MIR3142HG
TNFAIP3
NFKBIA
TNIP3
KRT81
TF
D
Wound Closure CF vs Wound Closure Flag CF
SOD2
SLC11A2
VNN1
ZC3H12A
LYN
CXCL1
ENPP6
CCL20
SGPP2
LRRC55
VNN3
TNFAIP2
CXCL3
NFKBIA
SLC26A4
EPS8L3
CSF3
TCIM
MIR3142HG
CXCL5
RAB37
E
Not Wounded Flag NCF vs Wound Closure Flag NCF
FGFBP1
S100A2
FST
DUSP7
PRR11
CDC6
ACOT7
S100A16
SRM
HASPIN
IL36A
CCDC28A
CREBRF
SPRR2F
KLHL24
CRCT1
F
Not Wounded Flag CF vs Wound Closure Flag CF
FGFBP1
S100A2
PROCR
DUSP7
PRR11
FST
TMC7
SRM
CCND1
S100A16
CKAP2L
IL18
DKK1
SH3BGRL3
MSN
ACOT7
FSTL3
COTL1
RPL22L1
RP5−1024G6.5
LINC01770
BCL2L11
ISYNA1
G
Not Wounded Flag NCF vs Not Wounded Flag CF
IL36A
SPRR2F
ZFHX4
FAM107A
RTKN2
ADAMTS9
MXD1
FMO3
LMCD1
IL36RN
SOX9
ERMN
HEPHL1
NR1D1
SPRR2A
ENPP2
SIX3
SLC22A16
CHST4
RP11−510M2.5
LL21NC02−1C16.2
H
Wound Closure Flag NCF vs Wound Closure Flag CF
SLC22A16
ZFHX4
FMO3
SIX3
TNNI2
SYT8
ZNF726
FAM107A
CTSV
RNASE1
DUOXA2
ALPL
− Log10 P
Log2 fold change
Downregulated
Unregulated
Upregulated

A

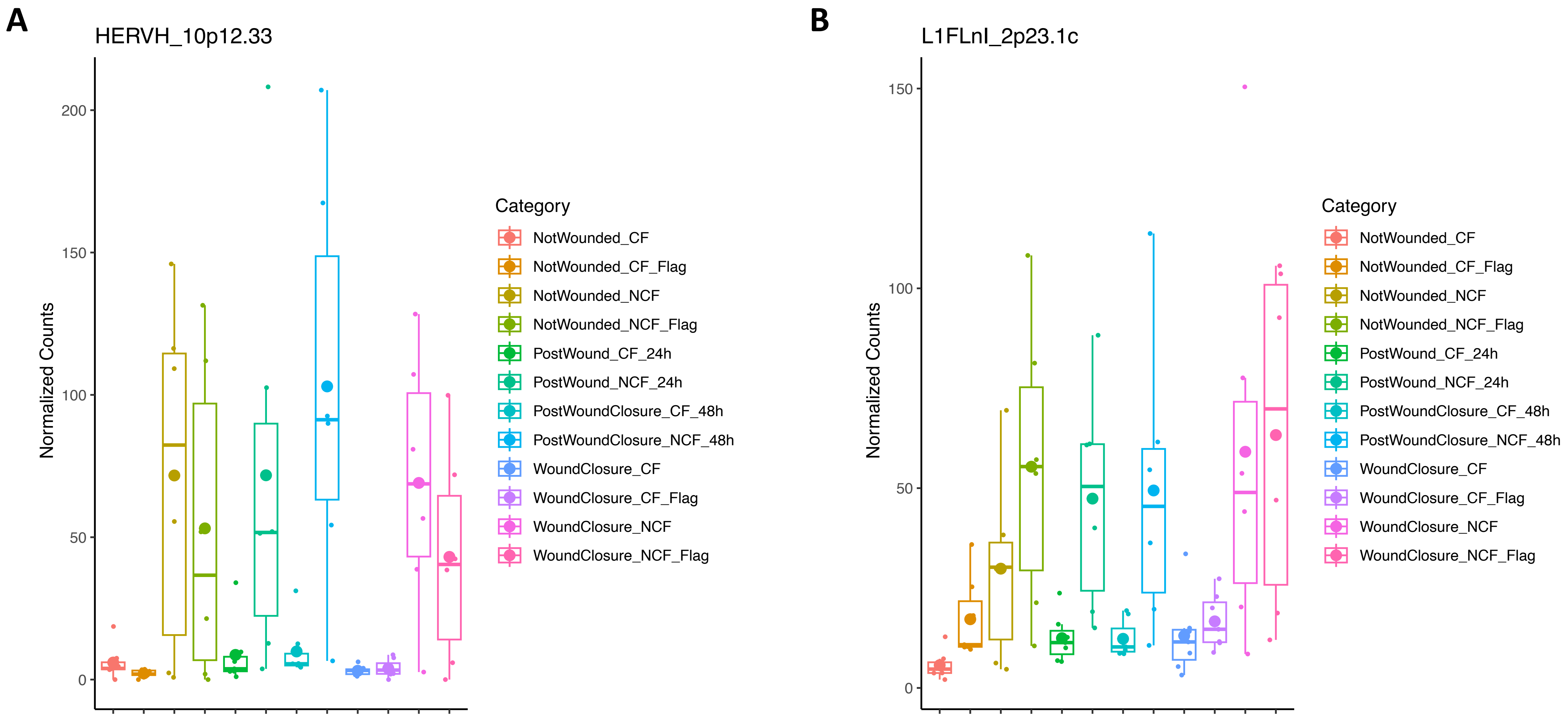

A
HERVH_10p12.33
Normalized Counts
200
150
100
50
0
B
L1FLnI_2p23.1c
Normalized Counts
150
100
50
0
Category
NotWounded_CF
NotWounded_CF_Flag
NotWounded_NCF
NotWounded_NCF_Flag
PostWound_CF_24h
PostWound_NCF_24h
PostWoundClosure_CF_48h
PostWoundClosure_NCF_48h
WoundClosure_CF
WoundClosure_CF_Flag
WoundClosure_NCF
WoundClosure_NCF_Flag
